# Design of a Remote Real-Time Monitoring System for Multiple Physiological Parameters Based on Smartphone

**DOI:** 10.1155/2019/5674673

**Published:** 2019-11-19

**Authors:** Noman Q. Al-Naggar, Husam Mohammed Al-Hammadi, Adel Mohammed Al-Fusail, Zakarya Ali AL-Shaebi

**Affiliations:** Department of Biomedical Engineering at Faculty of Engineering, University of Science and Technology, Sana'a, Yemen

## Abstract

**Background:**

Utilization of the widely used wearable sensor and smartphone technology for remote monitoring represents a healthcare breakthrough. This study aims to design a remote real-time monitoring system for multiple physiological parameters (electrocardiogram, heart rate, respiratory rate, blood oxygen saturation, and temperature) based on smartphones, considering high performance, autoalarm generation, warning transmission, and security through more than one method.

**Methods:**

Data on monitoring parameters were acquired by the integrated circuits of wearable sensors and collected by an Arduino Mega 250 R3. The collected data were transmitted via a Wi-Fi interface to a smartphone. A patient application was developed to analyze, process, and display the data in numerical and graphical forms. The abnormality threshold values of parameters were identified and analyzed to generate an autoalarm in the system and transmitted with data to a doctor application via a third-generation (3G) mobile network and Wi-Fi. The performance of the proposed system was verified and evaluated. The proposed system was designed to meet main (sensing, processing, displaying, real-time transmission, autoalarm generation, and threshold value identification) and auxiliary requirements (compatibility, comfort, low power consumption and cost, small size, and suitability for ambulatory applications).

**Results:**

System performance is reliable, with a sufficient average accuracy measurement (99.26%). The system demonstrates an average time delay of 14 s in transmitting data to a doctor application via Wi-Fi compared with an average time of 68 s via a 3G mobile network. The proposed system achieves low power consumption against time (4 h 21 m 30 s) and the main and auxiliary requirements for remotely monitoring multiple parameters simultaneously with secure data.

**Conclusions:**

The proposed system can offer economic benefits for remotely monitoring patients living alone or in rural areas, thereby improving medical services, if manufactured in large quantities.

## 1. Introduction

The practice of remotely monitoring physiological parameters has become prevalent. Smartphones and wearable sensors (WS) are widely available and can provide real-time monitoring of critical parameters for healthcare providers and patients. Thus, integrating and combining WS and smartphone technology (WSST) in a system can reduce the challenges in monitoring life parameters of patients with complex health conditions regardless of their location (e.g., remote or rural areas) [1–3]. The use of WSST can likewise improve telemedicine and healthcare services and provide progressive services to patients with chronic conditions [1, 4].

Numerous innovations have been developed for real-time monitoring and/or store-and-forward telemedicine services using ubiquitous connectivity tools and simple mobile phone or WS applications. WSST has developed over time owing to the creation of various built-in applications and communication tools, such as GPS and third-generation (3G) and fourth-generation phone network Internet [5, 6]. WSST development has been accompanied by the increasing number of smartphone users, which was predicted to grow from 2.1 billion in 2016 to approximately 2.5 billion in 2019, as well as the growing number of applications being developed for real-time monitoring and health diagnosis [3, 7].

Remote monitoring systems have improved progressively to meet the needs of the elderly as well as to reduce the number of deaths from chronic diseases, such as cardiac arrhythmia, high blood pressure, and diabetes [8]. Therefore, numerous studies have been conducted to monitor multiple physiological parameters responsible for such diseases [1, 9–14]. However, other studies have focused on developing WSST to monitor a specific disease [15–18].

Several studies have emphasized the use of developed WSST in health applications given the positive static data measurement characteristics of WSST, such as reliability and accuracy in continuous or real-time monitoring [19–21]. Other studies have focused on the disadvantages of WSST, such as high power consumption, the generation of false alarms, long-term health monitoring efficiency, and large-scale utilization [1, 3, 20]. A few studies have also discussed a combination of mobile technologies or monitoring issues [3, 22, 23].

The limitations of such designed systems include measurement of a single parameter, analysis, and data transmission and reception method/time [15–18]. Improvements in this field have mostly tried to overcome these limitations in ambulatory applications, specifically, the monitoring of only one vital parameter, battery life, cost-effectiveness, monitoring functions, a monomethod for warning transmission, and data security.

The current study integrates the specialized WSST to design a remote real-time monitoring system for multiple physiological parameters based on smartphones with developed health applications that can monitor and display measured critical parameters, including an electrocardiogram (ECG), heart rate (HR), respiratory rate (RR), blood oxygen saturation (SpO_2_), and temperature. The proposed system should meet main requirements such as sensing, processing, displaying, and real-time transmission and should have the capability to generate an autoalarm based on the analysis threshold values of multiple monitoring parameters. Moreover, it should ensure the dispatch of warning messages via two transmittal methods, namely, short message service (SMS) and the Internet, and identify a patient's location via GPS. The system should also meet auxiliary requirements such as compatibility, comfort, low power consumption and cost, and small size. Furthermore, the developed application should enable users to record, save, and transmit real-time data in video and text forms.

In this study, a WS acquires the body data, which are first sent to an Arduino Mega 2560 R3 and then to a smartphone through a Wi-Fi interface. The data are collected in a smartphone using a developed patient application that analyzes, processes, and displays the data before transmitting them to the developed doctor application. The patient application consists of two working modes. The first mode continuously transmits data, while the second mode transmits data only when abnormality is detected. Thus, the second mode saves phone or device power as well as the time of doctors/operators. The application prompts an autoalarm when an abnormal value is detected based on a previously identified threshold and sends a warning message to the doctor application (doctors/operators). The developed application is designed with adequate security to protect patient information. A special power bank is also designed to ensure the long feeding power of the system and smartphone.

System performance and reliability are evaluated for accuracy measurement, power consumption test against time, and average time delay. The average accuracy measurement of the collected parameters is 99.26%, and the system achieves low power consumption against time (4 h 21 m 30 s) for feeding measured circuits. Moreover, the result shows that the average time delay of data transmission to the doctor application via Wi-Fi is 14 s, whereas that via a 3G mobile network is 68 s.

The results demonstrate the reliability and acceptability of the system as well as the achievement of main and auxiliary requirements. Therefore, the current system is recommended not only for rural areas, particularly in developing countries, but also for hospitals and specific health centers and to provide first aid, primary diagnosis, and treatment. The system will also offer economic benefits if manufactured in a large scale.

## 2. Materials and Methods

**Figure 1:**
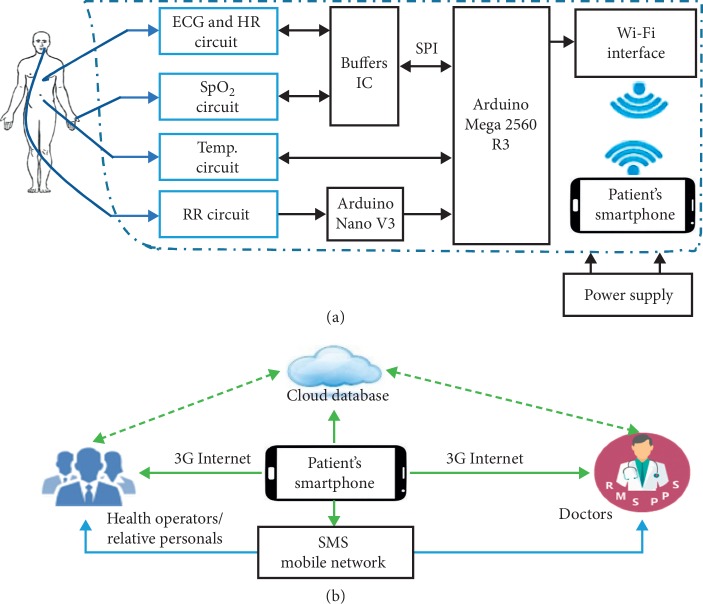
Overview of designed system architecture: (a) circuit diagram connectivity between WS, Arduino board, Wi-Fi module, and smartphone; (b) overview of data transmission network.

The proposed design was achieved through a combination of WS circuits and smartphone technologies via an Arduino circuit (as shown in Figure 1). The WS circuits acquired and computed the body data using an Arduino, which performed primary data collection. An electronic interface connected the Arduino circuits to the smartphone application to monitor, analyze, process, and transmit the data. The data were ensured and secured for intended persons only.

Figure 1 shows an overview of the designed system architecture, in which Figure 1(a) shows a block diagram of the hardware components and their connectivity sequence and Figure 1(b) demonstrates the data transmission network of the monitoring parameters.

### 2.1. Hardware Components

All hardware components were carefully selected to meet the requirements of the proposed design, namely, low power consumption, suitability for ambulatory applications, accuracy, reliability, affordability, and availability. Auxiliary requirements such as easy handling, comfort, minimal weigh, and long-term battery power (power consumption) were also considered, as these features could solve and overcome the limits and challenges of this field [3]. The hardware components are elucidated as follows.

#### 2.1.1. ECG and HR Circuits

These circuits were used to acquire the first measuring parameter (i.e., an ECG) from which the second parameter (i.e., HR) was calculated using a MAX30003 circuit [24], which reduces movement artifacts during continuous monitoring and is common in telemetry monitoring [8].

The ECG circuit removed motion artifacts using an instrumentation amplifier that has a two-pole active antialiasing filter with a 600 Hz−3 dB frequency. The high-pass filter options included a first-order infinite impulse response (IR) Butterworth filter with a 0.4 Hz corner frequency, which was selected to correspond to ambulatory applications. The low-pass filter options included a 12-tap linear phase (constant group delay) finite IR filter with a 40 Hz corner frequency. The amplification process in this study used 20 V/V.

The raw data of the ECG signals were saved in the memory of the ECG circuit and then sent as a sequence to the Arduino (Mega 2560 R3) using a DM74LS125A integrated circuit (IC) through a high-speed interface to prevent interference between the data of the ECG signal and other signals.

*(1) HR Extraction*. HR was defined by calculating the *R*-*R* duration/interval time among QRS complexes of consecutive ECG waveforms within 1 minute intervals, where *R* was the first upward deflection wave after the P wave, the QRS complex was a series of waveforms following the P wave in the ECG waveforms, and the R-R interval was the elapsed time between two consecutive R waves [25, 26].

In this work, R waves were extracted by an Android program from the recorded ECG waveforms as the maximum. Next, identical maximum points (*R* − *R* interval,  ms) were calculated, averaged, and divided into 1 minute intervals (60 × 1000 ms). Hence, HR was calculated in beats per minute (bpm) as follows:

(1) $$\begin{array}{c} \text{HR}\left(\text{bpm}\right)=\frac{60*1000}{R-R\text{ interval}\left(\text{ms}\right)}. \end{array}$$

#### 2.1.2. SpO_2_ Circuit

The SpO_2_ signal was acquired by a finger probe using an AFE 4490 from Texas Instruments [27], which utilized a pulse oximeter technique (light-emitting diodes). The signal presented a voltage to the 22-bit analog-to-digital converter (ADC), which was fed to a data processor to digitize and send the display signal.

#### 2.1.3. Temperature Circuit

The body temperature signal was acquired through skin temperature using a MAX30205 temperature sensor, which provided a digital output using an ADC and operated within the 0°C to +50°C temperature range. The completed temperature reading operation was updated for a new temperature measurement. During this process, changes in temperature were discounted until the peer reading was completed. The updated temperature register was sent to the Arduino Mega 2560 R3 to process and display the signal.

#### 2.1.4. RR Circuit

RR was acquired by a circuit that consisted of a 10 K-negative temperature coefficient thermistor fitted into a nebulizer mask with a voltage divider configuration. Thermistor resistance decreased during exhalation owing to comparatively hot air and increased during inhalation. The obtained signal from resistance was converted into a voltage and fed into a 0.0884–0.8942 Hz bandpass filter. The output of the filter was amplified 100 times and sent to the Arduino Mega 2560 R3 through an Arduino Nano, as shown in Figure 1.

#### 2.1.5. Power Supply Circuit Bank

The power supply circuit was designed to meet the requirements of the power bank for the proposed system, such as low cost and effective long-time consumption.

**Figure 2:**
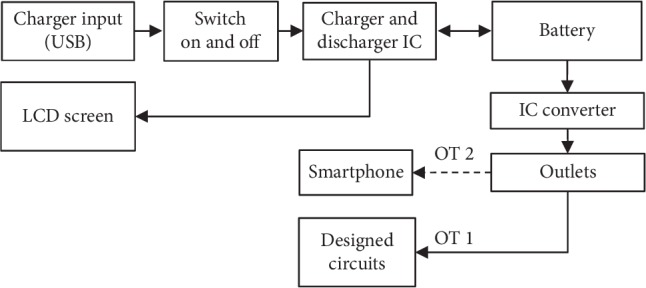
Block diagram of the power supply circuit bank.

Figure 2 illustrates the components of the designed circuit bank, which consisted of a charger/discharger IC (TP4056 chip), an IC converter, an LCD screen, and a lithium-ion battery. A TP4056 chip was used to control the charging and discharging of the battery, which was supplied by a control switch through an “on” and “off” function. A DC-DC boost IC converter chip was used to cater to a stable 5 V DC supply at outlets to ensure a supply of not less than 3.7 V DC from the battery. The LCD screen showed the percentage of the remaining capacity and the working status. A USB outlet 2 (OT 2) was used as an option for charging smartphones (if needed), and an outlet 1 (OT 1) was used to feed the circuits via constant current and voltage (5 V). The actual capacity of the battery was 3678 mAh, and actual recharging time was 1 h 50 m 15 s. The designed features/specifications of the power supply circuit bank are demonstrated in Table 1.

#### 2.1.6. Arduino Mega 2560 R3

An Arduino Mega 2560 R3 was selected owing to its memory capacity, multiple and various input/output pins, data-processing speed, various WS connection modes, and simple computer connection via a USB cable. Moreover, it included an option to send signals wirelessly or via a USB cable.

### 2.2. Patient Data Collection and Transmission

#### 2.2.1. Patient Data Collection

The acquired data from multiple WS were collected in the Arduino Mega 2560 R3, which was the primary platform for data collection and preparation for transmission to Android devices. The Arduino was connected to a tablet or a smartphone via a USB cable and a computer to display the acquired data during the tests. However, this capability enabled monitoring parameters in sideway locations.

#### 2.2.2. Patient Data Transmission

Patient data were transmitted from the Arduino Mega 2560 R3 to an Android smartphone using a Wi-Fi ESP8266 circuit through serial communication (RX/TX lines), which was capable of either hosting an application or offloading all Wi-Fi networking functions from another application processor. The Wi-Fi ESP8266 circuit was used rather than Bluetooth owing to the former's standby power consumption of <1.0 mW and waking-up and packet transmission of <2 ms and capability to send a variety of data.

A smartphone's Wi-Fi was forced to fast switch (833 *μ*s) between two operations automatically to receive and transmit data to the doctor application. The system did not lose its capability for real-time transmission because of the delay from switching.

### 2.3. Android Health Application Design

The application design considered features that help increase the probability of saving patient lives as well as modern attributes, thereby presenting advantages over other designs in recent studies [2, 21]. The current application consisted of patient and doctor applications.

#### 2.3.1. Patient Application

A health application was created in the proposed system in the Android studio interface to simplify the procedures of the intended features. The Android applications were written using the Java programming language. The primary data collected in the patient application were converted into integer values (secondary data) and compared with the threshold values of each parameter. The application continuously scanned for updating parameter values simultaneously and visualized the values in the adaptation window of the smartphone. The application worked in the following modes:

- The first mode monitored and displayed parameter values simultaneously in real time through a smartphone, with a possibility of transmitting these values to intended trends.
- The second mode transmitted data when abnormal/threshold values were detected. That is, the system monitored and sensed parameter value abnormalities and then transmitted these data to intended trends.

Selection of the second mode helped arrange/save time for personnel working with related proposed systems. Moreover, this mode saved a considerable amount of energy; thus, it provided more advantages than those in previous works [1, 2, 8, 10, 14]. The developed application also included the following features:

- Provided a platform for monitoring and displaying measured parameters based on primary analysis and diagnosis
- Saved recorded data with respect to time to review activities during movements and exercises
- Transmitted data in video (graphic and numerical data) or numerical forms to responsible individuals (doctor application)
- Offered more than one option for monitoring and transmitting data
- Sent patient locations via GPS using Wi-Fi or 3G as well as a warning to other personal operators via mobile or/and Internet networks using SMS or/and WhatsApp, respectively

#### 2.3.2. Doctor Application

The second component of the health application was created in Android and provided a screen window to show the transmitted data from the patient application. This component permitted doctors or responsible persons from an insurance company or medical center to monitor a patient's situation and provide first aid and diagnosis for critical cases. The web portal requires a user name and ID/password to protect privacy. The web interface provided data in video form (graphic and numerical data) recorded from the patient application and/or numerical data for multiple patients to display on smartphone/Android devices.

#### 2.3.3. Application User Management

**Figure 3:**
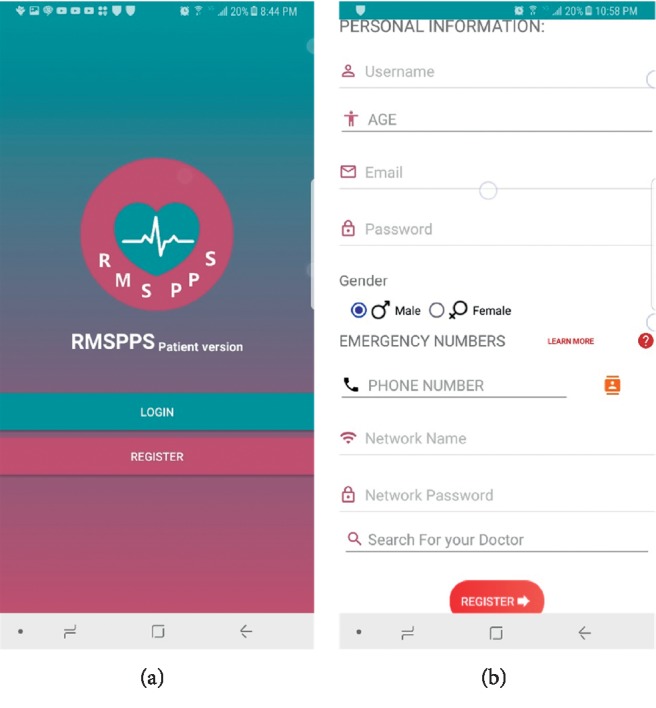
First windows of the patient application: (a) log-in and registration screen option; (b) patient information registration screen (e.g., name, age, gender, and the emergency number of the intended person).

**Figure 4:**
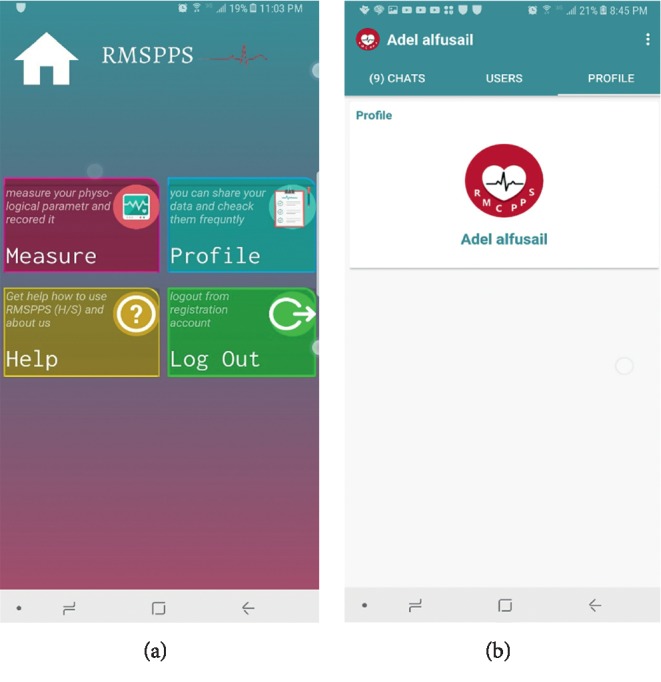
The menu interface of the patient application: (a) home screen of the patient application; (b) the patient account page.

The application interfaces and icons were designed in a simplified manner to be managed and used easily by anyone, as shown in Figure 3. The menu interface of the patient application is shown in Figure 4.

### 2.4. Autoalarm System

The designed system generated an autoalarm in the two working modes of the health application when it sensed abnormalities in one or more of the monitored parameters and would transmit data using two warning methods.

#### 2.4.1. Wi-Fi/3G Warning Method

**Figure 5:**
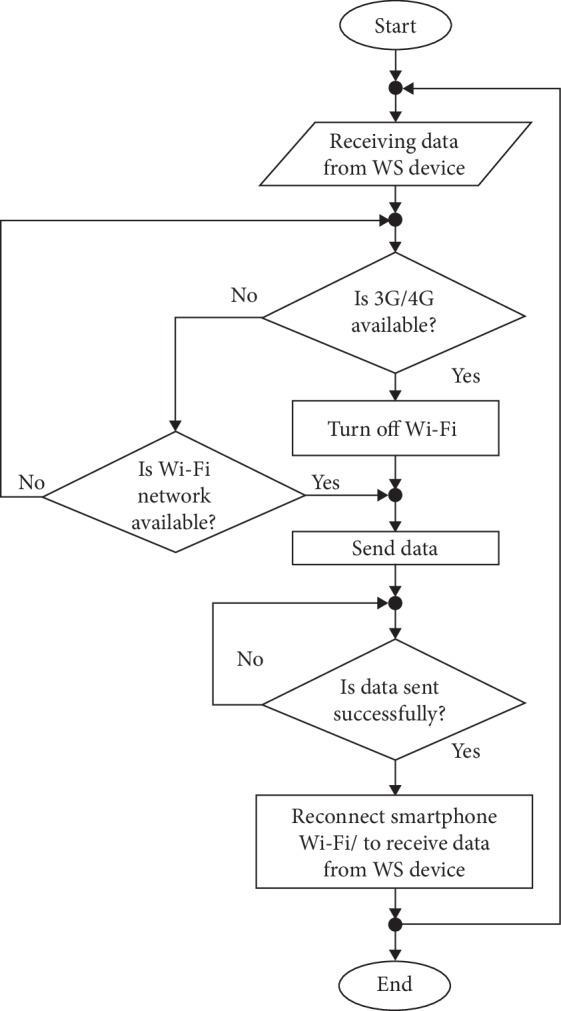
Smartphone Wi-Fi connection-reconnection sequence.

This method was used in both application modes to transmit data to the web interfaces of intended trends. The 3G network was dominant in smartphones for transmitting and receiving data via Wi-Fi from the Arduino owing to the default Wi-Fi system, as shown in Figure 5.

Wi-Fi was reconnected automatically for 833 *µ*s to receive data from the Arduino to send to the doctor application. In case of available Wi-Fi NAN, such as in rural areas and locations far from health facilities, the system used 3G Internet provided by a mobile phone network. The autoalarm generated through the system was received on the web interface and perceived through sound and vibration to notify doctors or operators with identified locations via GPS.

#### 2.4.2. Mobile Network Warning Method

This method was used to send SMS warning messages to centers such as RMSPPS servers, families, or doctors. The message was shown as “I have detected an abnormality condition; for more details, visit your account on the doctor application.” Phone numbers of the operator's server/insurance company and doctors were identified previously in the system.

**Figure 6:**
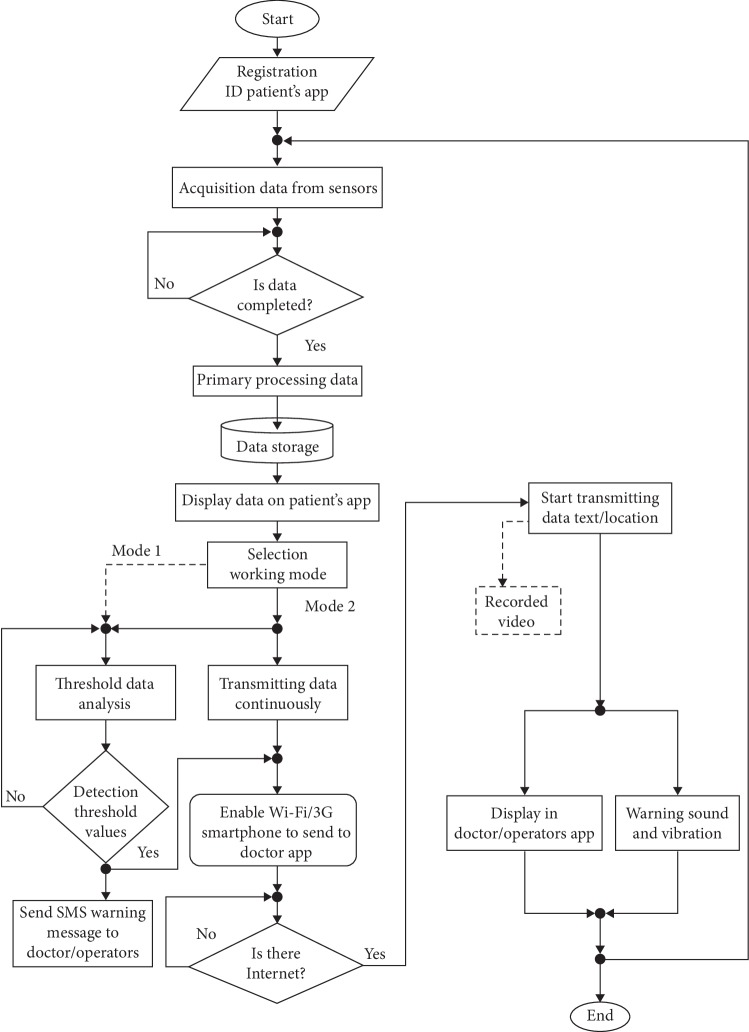
Flowchart of the working mechanism of the designed system.

Figure 6 shows the sequence of the application working mechanism, threshold value identification, and data transmission. The default mode in the application was mode 1, whereas mode 2 in the dashed line was considered as a user option.

### 2.5. Determining Threshold Values

The autoalarm feature of the proposed system was based on the threshold value determination of monitored physiological parameters, such as HR, which reflects certain cases of ECG abnormalities and is considered as an indicator of a motion function [28]. In this study, HR was extracted and calculated from ECG waveforms on the basis of an algorithm proposed in [25], and the HR threshold values were determined based on works [13, 26].

RR threshold values have been defined in different ranges depending on the acquisition method and age of a patient [29] and are considered as an indicator of various symptoms, such as cardiac arrest, coughing, decreased alertness, poor feeding, grunting, and fever [30–32]. The mean observed RR was 14.2 (±4.17 precision (SD)) breaths per minute for adults [29], which was less than the mean RR 15.1 (±4.05 SD) breaths per minute measured by respiratory inductive plethysmograph [32]. In this study, the normal RR for monitoring elderly and adult subjects (between the ages of 20 and 50 years) at rest was from 12 breaths per minute to 16 breaths per minute. The common RR abnormality limits are shown in Table 2 [30, 33].

Temperature and SpO_2_ estimation values were determined as ranges that have been defined for pathologies [1, 13]. Table 2 shows a summary of the threshold values of intended parameters.

### 2.6. System Test

The proposed system was subjected to numerous tests to determine the accuracy and reliability of remote monitoring and data transmission in real time. The system primarily evaluated the achievement of designed and functional requirements at each implementation step. Quality performance of the system was measured by calculation accuracy. Accuracy was determined by the agreement between the measured (experimental) values of the proposed system and the true value of the qualified equipment (patient monitor model TR6628-9500, Eastern Europe Co.) in the biomedical engineering laboratories at the University of Science and Technology, Yemen. Equation (2) demonstrates the percent error calculation, and equation (3) shows the percent accuracy calculation:

(2) $$\begin{array}{c} \text{percent error}=\frac{\text{measured value}-\text{true value}}{\text{true value}}*100, \end{array}$$

(3) $$\begin{array}{c} \text{percent accuracy}=100\%-\text{percent of error}. \end{array}$$

The accuracy measurement of each sensor was calculated by averaging the obtained accuracy values of five measurement processes. Then, the total accuracy of the entire WS was calculated as the average of the total WS measurement accuracy, as shown in Tables 3 and 4.

System reliability was tested and held in different time periods. Power consumption for long-term use was evaluated through battery life charge time (LCT) for three cases, namely, supplying only the working circuits, charging the circuit and smartphone simultaneously (versatility), and supplying only the smartphone (an auxiliary), as shown in Table 5. Likewise, reliability of the system for data transmission delay time was checked with Wi-Fi and 3G. The system held seven connection/disconnection trials, considering several connection conditions, such as weak Internet, coverage area, and cloud server type (see Table 6).

## 3. Results

The obtained results illustrated the achievement of the current system in monitoring critical parameters related to common diseases, such as HR, ECG, SpO_2_, and temperature, which were measured using a combined WS and Arduino circuits and a developed application in Android devices, such as smartphones. The patient application met the requirements of receiving, processing, analyzing, and transmitting data to intended trends as well as displaying them in the doctor application/web interface. The results are organized to show the main achievements.

**Figure 7:**
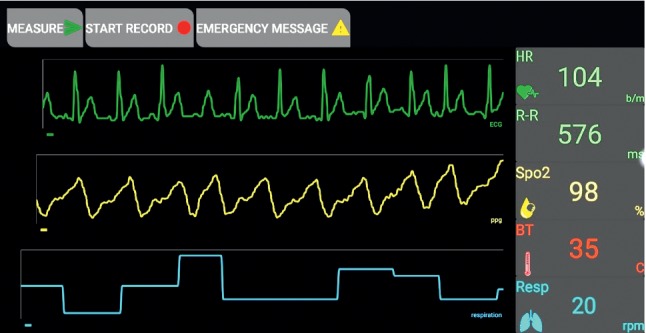
Interface display of monitoring parameters (application display).

Figure 7 shows the interface display for monitoring data, which was represented in graphic and numerical forms. These features promoted readability for healthcare personnel, such as patients, doctors, nurses, and operators. The exterior interface was designed like a patient's monitor, reflecting the same functions and information but through a portable device.

The results revealed the success of the autoalarm generation system in case of abnormalities and in sending a warning SMS via the system to the doctor application through sound and vibration as well as a written flag to attract a doctor's attention.

**Figure 8:**
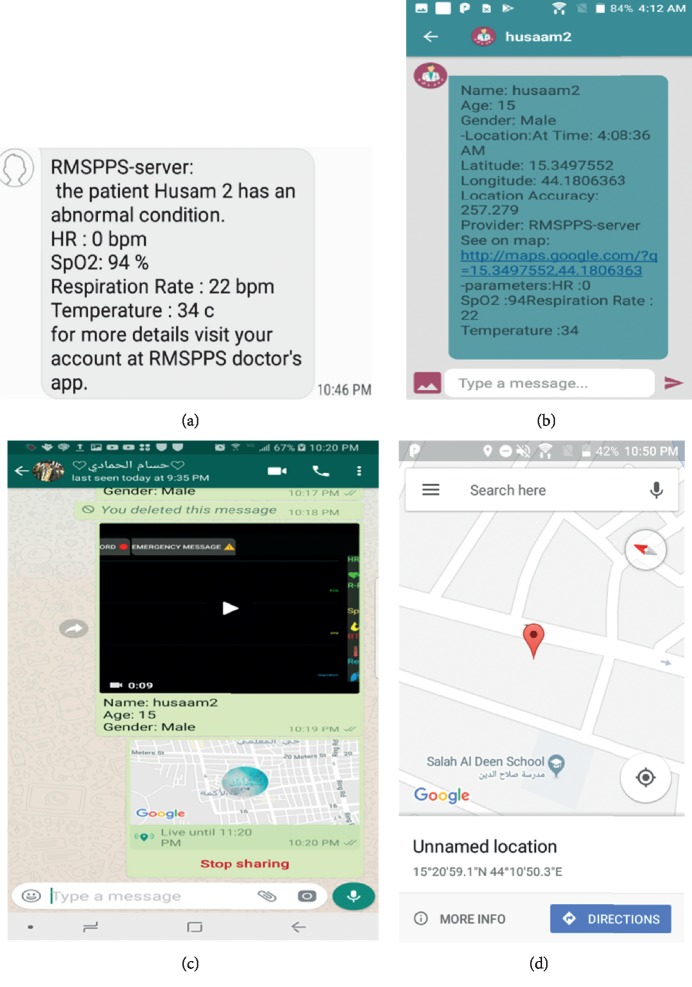
Several capabilities of the designed system: (a) example of receiving an SMS; (b) transmitted data in the doctor application; (c) receiving a video recording and a message in WhatsApp; (d) sending a patient's location to a doctor/operator.

Figure 8 shows several capabilities of the designed system, in which Figure 8(a) illustrates an example of an SMS received via a mobile network. Therefore, the system was useful for tracking patients, particularly when Internet connection was unavailable. The system transmitted data and/or video recordings of acquired abnormal cases to doctors/operators and displayed them via the doctor application and WhatsApp, as depicted in Figures 8(b) and 8(d).  Figure 8(c) demonstrates the system's capability to determine locations via GPS.

Tables 3 and 4 show the results of the accuracy measurement evaluation by comparing the values of our system with a standard device. The accuracy measurement was calculated as the average accuracy of each measured parameter, and all the accuracy measurements were averaged as the accuracy measurement of the entire system (99.25%).

The results of the power consumption test against time showed sufficient LCT in the case of supplying the circuits (4 h 21 m 30 s). However, in the versatility case, LCT was low and approximately 45% of time, as shown in Table 5.

Table 6 demonstrates the results of the time needed to connect stages and transmit data through the system for Wi-Fi and 3G. The average time for data transmission to the doctor application was 18 s via Wi-Fi and 70 s via 3G. The average time delay was less (14 s) with a Wi-Fi network compared with a 3G network (68 s). Therefore, the system can serve its purpose, and the alarm response time depended on the smartphone model and Internet speed.

**Figure 9:**
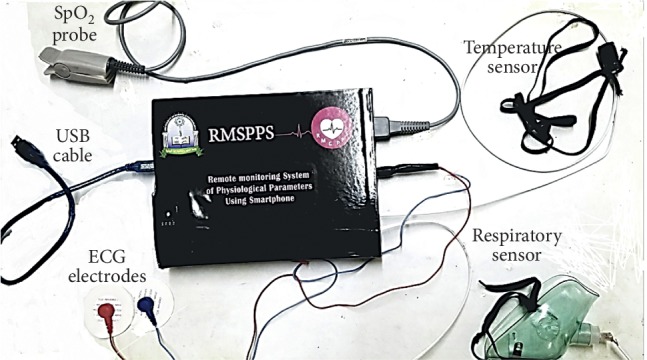
Final external view of the designed system.

The final specifications and features of our system are concluded in Table 7, and the final design is shown in Figure 9.

## 4. Discussion

The results of the proposed system appear clearly and correctly without interference in the monitored parameter data in the patient and doctor applications.

The provided features in the interface display in graphic and numerical forms (Figure 7), distinguish the proposed system, and overcome the challenges in previous works in this field [1, 2, 8, 10, 21]. Moreover, the system includes other capabilities that improve primary diagnosis to offer first aid, particularly for simultaneous critical cases of multiple patients. These capabilities include generating an autoalarm and transmitting data in multiple forms. The system also exhibits general features, such as sending data to social media (WhatsApp) and determining patient location via GPS.

The system demonstrates a satisfactory time delay (14 s) for data transmission via Wi-Fi compared with the recent presented system (30 s) [1]. The time needed to transmit data via 3G is 68 s owing to the low speed used in the tested area, which can be improved. A 3G mobile network can provide the proposed system with long real-time monitoring to achieve an extensive coverage area. The low and acceptable power consumption against time (4 h 21 m 30 s) is an important feature that distinguishes our system. Thus, the use of less time for transmitting data, with a potential for improvement, and the long operation time make our system superior to other systems in the related work in this field [1, 10, 14].

The results of the combined WSST and developed application meet the main requirements for remotely monitoring physiological parameters. These requirements include a remarkable achievement in design and efficiency improvement in terms of accurate real-time sensing, transmuting, and displaying as well as low power consumption. Moreover, the system achieves auxiliary requirements such as comfort, compatibility, ease of use, minimal weight, small size, and affordability, which are considered more than the stated requirements in such systems [3].

Despite its features and advantages, the system should be further developed and adapted for iPhone operating systems. That is, the application is compatible only with Android devices, specifically Android operating systems starting from version 5.1.1. A blood pressure monitoring parameter should be added to the current five parameters of the system for the full monitoring of critical cases. In this study, our emphasis on the use of Wi-Fi rather than Bluetooth for sending data to smartphones is justified owing to the former's data transmission speed and minimal power consumption. Thus, the two features distinguish the system and meet the essential requirements for real-time monitoring.

## 5. Conclusion

The use of WSST in a remote monitoring system can advance healthcare services, particularly for the elderly who live alone or in rural areas with limited access to medical services or institutions. In this study, a remote monitoring system is designed based on smartphones to perform real-time monitoring and to provide primary analysis, diagnosis, and treatment (i.e., first aid) simultaneously. The proposed system helps reduce the death rate from chronic and common diseases related to the monitoring of critical parameters, such as ECG-HR, SpO_2_, RR, and temperature.

The system also possesses features that render it superior to other systems in the field, such as application capabilities, option modes, autoalarm generation, alarm transmission via two methods, secured data transmission, and appropriate forms for displaying such data for multiple patients simultaneously. Moreover, the system meets the main and auxiliary requirements.

Consequently, the designed system presents a solution not only for rural areas in developing countries but also for all types of healthcare facilities. Furthermore, the system would be economically beneficial if manufactured in large quantities because it would lead to the development of widespread health service networks in developing countries as well as rural areas.

In the future, the system can add a blood pressure signal to the monitoring parameters, as it relates to critical cases. Moreover, the system will need to overcome its limits, such as adaptation to iPhone operating system devices, given the wide range of people using such devices.

## Figures and Tables

**Table 1:** Specifications of the designed power supply.

Parameter	Specifications
Battery type	Lithium ion
Capacity	4000 mAh
Connectivity	Two output ports (2 A feed system, 1 A USB to feed phone)
Voltage required input	USB 5 V, 2 A
Time required to recharge	1 hour 50 minutes
Battery life with full charge	4 hour 30 minutes

**Table 2:** Threshold values of monitoring parameters.

Parameter	Rhythm/pathology	Threshold values
SpO_2_ (%)	Normal	96 to 99
	Pulmonary or cardiovascular chronic diseases	Drop rapidly
	Acute respiratory failure	<90% + 3 to 4%

RR (breaths per minute (bpm))	Normal	12–16
	Cardiac arrest	≥27
	Lower respiratory tract infections	>24
	Tachypnea	>12–16
	Bradypnea	<12–16

Temperature (°C)	Normothermia or euthermia	37.0
	Fever	≥37.8
	Hypothermia	≤35.0

HR (beats per minute (bpm))	Normal	60 to 100
	Bradycardia	<60
	Tachycardia	>100

**Table 3:** Accuracy measurement of SpO_2_, HR, and ECG.

Subject	Parameter					
	SpO_2_ %		Heart rate (bpm)		ECG (*R*-*R* ms)	
	MV^*∗*^	TV^*∗*^	MV	TV	MV	TV
1	96	96	77	76	632	630
2	95	94	100	98	568	563
3	98	96	99	98	581	585
4	96	97	45	45	627	632
5	95	94	89	88	639	635
Average accuracy (%)	98.36		98.89		99.93	

^*∗*^MV, measured value; TV, true value.

**Table 4:** Accuracy measurement of RR and temperature.

Subject	Parameter			
	Respiratory rate (rpm)		Temperature (°C)	
	Measured value	True value	Measured value	True value
1	14	14	32	33
2	16	15	35	34
3	14	15	35	33.5
4	15	16	36	35
5	15	15	34.5	35.5
Average accuracy (%)	100.00		99.11	

**Table 5:** Power consumption test.

Test type	Description	Time
Battery LCT	Supplies the circuits only	4 h 21 m 30 s
	Supplies the circuits and smartphone (versatility)	2 h 15 m 20 s
	Supplies the smartphone only	3 h 50 m 05 s

**Table 6:** Performance test of transmission time.

Performance parameter	Wi-Fi	3G
Average connecting time (s)	71	117
Average transmitting time to doctor app (s)	18	70
Average time loss ratio (s)	4	2
Average time delay (s)	14	68

**Table 7:** Final specifications of the designed system.

WS/parameter	Specifications
General	(i) Rechargeable battery
	(ii) Compatible with most android devices
	(iii) Small size, portable, and easy to use
	(iv) Comfortable for adult and old patients
	(v) Multiple parameters: ECG, HR, SpO_2_, temperature, and RR
	(vi) The ability of recording a video and data text of signals
	(vii) Sending autoalarm to the doctor/centers in different tools

ECG and HR	(i) ECG: single lead without the need for a third right-leg drive (DRL) electrode
	(ii) Heart rate detected by *R* to *R* distance
	(iii) Frequency range: 15.625 mHz up to 256 Hz
	(iv) ECG calibration: ±0.25 mV
	(v) Arrhythmia analysis: yes

SpO_2_	(i) Display: waveforms and digits
	(ii) Real-time display of PPG (photoplethysmogram)
	(iii) Measurement range: 1–100%
	(iv) Resolution: 1%
	(v) Accuracy: 2% (80–100%)
	(vi) Pulse rate range: 20–300 bpm

RR (respiratory rate)	(i) Method: air flow temperature
	(ii) Measurement range: 5∼50 rpm
	(iii) Accuracy: ±1 bpm
	(iv) Resolution: 2 bpm

Temperature	(i) Measurement range: 0∼50°C
	(ii) 0.1°C accuracy (37°C to 39°C)
	(iii) Resolution: 0.1°C

## Data Availability

The Arduino code and the Android applications that are used to support the findings of this study have been deposited in the RMSPPS repository on GitHub website (https://github.com/adelalfusail/RMSPPS).
